# Supplementary material for: Empirical methods for controlling false positives and estimating confidence in ChIP-Seq peaks
Source: BMC Bioinformatics. 2008 Dec 5;9:523. doi: 10.1186/1471-2105-9-523 (PMC2628906; doi:10.1186/1471-2105-9-523)
Supplement: Additional File 1 — A variety of html documents from the USeq web site detailing the available applications, their best usage, output file type descriptions, command line menus, etc. [file 1471-2105-9-523-S1.zip › USeqUserGuides/JavaDoc.zip!/JavaDoc/trans/roc/RocWindow.html]

RocWindow


|  |  |  |
| --- | --- | --- |
| **Overview** \| **Package** \| **Class** \| **Use** \| **Tree** \| **Deprecated** \| **Index** \| **Help** | |  |
| **PREV CLASS**   **NEXT CLASS** | **FRAMES**    **NO FRAMES**     **All Classes** |  |
| SUMMARY: NESTED \| FIELD \| CONSTR \| METHOD | DETAIL: FIELD \| CONSTR \| METHOD |  |


---


## trans.roc Class RocWindow

```
java.lang.Object
  trans.roc.RocWindow
```

**All Implemented Interfaces:**: java.io.Serializable, java.lang.Comparable

---

``` public class RocWindow extends java.lang.Object implements java.lang.Comparable, java.io.Serializable ```

For holding information about a region for ROC analysis.

**See Also:**: Serialized Form

---

**Constructor Summary**

`RocWindow(java.lang.String chromosome,
int start,
int end,
double score)`


| **Method Summary** | |
| --- | --- |
| `int` | `compareTo(java.lang.Object obj)` |
| `java.lang.String` | `getChromosome()` |
| `int` | `getEnd()` |
| `int` | `getMiddle()` |
| `double` | `getScore()` |
| `int` | `getStart()` |
| `int` | `getType()` |
| `boolean` | `intersects(RocWindow other)` |
| `static void` | `printSGRWindows(RocWindow[] w)` |
| `void` | `setChromosome(java.lang.String chromosome)` |
| `void` | `setEnd(int end)` |
| `void` | `setMiddle(int middle)` |
| `void` | `setScore(double score)` |
| `void` | `setStart(int start)` |
| `void` | `setType(int type)` |
| `java.lang.String` | `simpleSummaryLine()`             Returns a binding region line, tab delimited: chrom, start, end, score |

**Methods inherited from class java.lang.Object**

`equals, getClass, hashCode, notify, notifyAll, toString, wait, wait, wait`

**Constructor Detail**

### RocWindow

```
public RocWindow(java.lang.String chromosome,
                 int start,
                 int end,
                 double score)
```


**Method Detail**

### compareTo

```
public int compareTo(java.lang.Object obj)
```

:   **Specified by:**: `compareTo` in interface `java.lang.Comparable`

---


### intersects

```
public boolean intersects(RocWindow other)
```

---


### simpleSummaryLine

```
public java.lang.String simpleSummaryLine()
```

:   Returns a binding region line, tab delimited: chrom, start, end, score

---


### printSGRWindows

```
public static void printSGRWindows(RocWindow[] w)
```

---


### getChromosome

```
public java.lang.String getChromosome()
```

---


### setChromosome

```
public void setChromosome(java.lang.String chromosome)
```

---


### getEnd

```
public int getEnd()
```

---


### setEnd

```
public void setEnd(int end)
```

---


### getMiddle

```
public int getMiddle()
```

---


### setMiddle

```
public void setMiddle(int middle)
```

---


### getScore

```
public double getScore()
```

---


### setScore

```
public void setScore(double score)
```

---


### getStart

```
public int getStart()
```

---


### setStart

```
public void setStart(int start)
```

---


### getType

```
public int getType()
```

---


### setType

```
public void setType(int type)
```


---


|  |  |  |
| --- | --- | --- |
| **Overview** \| **Package** \| **Class** \| **Use** \| **Tree** \| **Deprecated** \| **Index** \| **Help** | |  |
| **PREV CLASS**   **NEXT CLASS** | **FRAMES**    **NO FRAMES**     **All Classes** |  |
| SUMMARY: NESTED \| FIELD \| CONSTR \| METHOD | DETAIL: FIELD \| CONSTR \| METHOD |  |


---
